# Breast Cancer-Derived Extracellular Vesicles: Characterization and Contribution to the Metastatic Phenotype

**DOI:** 10.1155/2015/634865

**Published:** 2015-10-27

**Authors:** Toni M. Green, Mary L. Alpaugh, Sanford H. Barsky, Germana Rappa, Aurelio Lorico

**Affiliations:** ^1^Comprehensive Community Cancer Center, Roseman University College of Medicine, Las Vegas, NV 89135, USA; ^2^Memorial Sloan Kettering Cancer Center, New York, NY 10065, USA

## Abstract

The study of extracellular vesicles (EVs) in cancer progression is a complex and rapidly evolving field. Whole categories of cellular interactions in cancer which were originally presumed to be due solely to soluble secreted molecules have now evolved to include membrane-enclosed extracellular vesicles (EVs), which include both exosomes and shed microvesicles (MVs), and can contain many of the same molecules as those secreted in soluble form but many different molecules as well. EVs released by cancer cells can transfer mRNA, miRNA, and proteins to different recipient cells within the tumor microenvironment, in both an autocrine and paracrine manner, causing a significant impact on signaling pathways, mRNA transcription, and protein expression. The transfer of EVs to target cells, in turn, supports cancer growth, immunosuppression, and metastasis formation. This review focuses exclusively on breast cancer EVs with an emphasis on breast cancer-derived exosomes, keeping in mind that breast cancer-derived EVs share some common physical properties with EVs of other cancers.

## 1. Introduction

Breast cancer is the most prevalent type of cancer in women [1]. Although a multitude of treatment options are available [2–4], approximately one-third of women worldwide diagnosed with breast cancer still die from the disease, largely from metastasis, especially brain metastasis [5–8]. EVs have been hypothesized to have significant roles in breast cancer growth and metastasis and thus have been evaluated as potential avenues of new therapeutic intervention. EVs, including exosomes and MVs, are secreted in large quantities by cancer cells into the local microenvironment and premetastatic “niche” [9]. While both exosomes and MVs are small (usually < 1 *μ*m in diameter), these bilipid membrane-enclosed vesicular structures [10–13] have a distinct biogenesis: exosomes are generated through inward budding of an endosome resulting in a multivesicular body (MVB) which is released by subsequent fusion of the MVB with the plasma membrane [14–18], whereas MVs are released directly by budding from the cellular plasma membrane [15, 18, 19]. However, EVs including both MVs and exosomes have been proposed to enter target cells through multiple mechanisms, including ligand-receptor-mediated [20, 21], or lipid raft-mediated entry [22, 23], with EV fusion and uptake greatly influenced by surrounding pH levels [24]. Exosomes are typically smaller than MVs, being 100 nm or less in diameter, while the latter are > 100 nm in size. Some size overlap does exist, however [25–29]. Apoptotic bodies, another type of EV, are poorly characterized and not generally included within the general categories of exosomes or MVs and therefore will not be discussed in this review. This review will discuss breast cancer EV purification, composition, and effect on target cells within the tumor microenvironment to promote cancer growth and metastasis, as well as their possible use as drug delivery vehicles of anticancer therapeutic drugs.

## 2. Breast Cancer EV Characterization and Proteomic Profiling

### 2.1. Methods of Isolation

There are several variations to the method of isolating MVs and exosomes from cell culture supernatants, but most involve differential centrifugation at various speeds to separate particles based on size and density. For EV isolation, cells are grown in media supplemented with exosome-depleted fetal bovine serum (FBS). The first step in purification involves a low-speed centrifugation at 300 ×g–500 ×g for 5–10 min to remove live cells [30–32]. The supernatant is then centrifuged at 1200 ×g–2000 ×g for 10–30 min to remove dead cells and apoptotic blebs [30, 33–35]. This is followed by centrifugation at 10,000 ×g for 30 min to remove cellular debris [30, 36]. To isolate the exosome and MV fractions, the supernatant then undergoes ultracentrifugation at 100,000–200,000 ×g for 60–120 min [30, 32, 36–38]. The supernatant at this point is discarded and the pellet, containing exosomes and MVs, is washed in phosphate buffered saline (PBS) and ultracentrifuged one final time [30, 36, 39, 40]. The PBS is gently decanted and the exosome/MV pellet is resuspended in a small volume of buffer [35, 41]. If human or mouse serum is used as starting material, an intermediate step using a 0.22 *μ*m filter can be employed to supplement the centrifugation steps, resulting in an enrichment in exosomes and small MVs [32, 38, 42]. These methods have been used to isolate MVs and exosomes from both cancer and noncancerous cell lines, including LNCaP prostate cancer, vascular smooth muscle cells (VSMCs), BV-2, HCMEC/D3, and several breast cancer cells (MDA-MB-231, MCF-7, and MDA-MB-435) [30, 31, 34, 43]. Exosomes purified using this methodology are suitable for liquid chromatography-mass spectrometry (LC-MS) analysis [30, 36]. However, in order to obtain a density-based purer fraction devoid of additional nonspecific proteins and protein aggregates, the exosomal/MV supernatant can be layered on a sucrose gradient prior to high-speed ultracentrifugation [33, 44]. It is really not possible to separate exosomes from MVs by strictly physical means.

### 2.2. Size Variation

Scanning electron microscopy (SEM) and transmission electron microscopy (TEM) have been used to determine the physical properties of EVs from many sources, including cancer and noncancerous cell lines, as well as from patients with a myriad of diseases. In general, exosomes are 30–100 nm in size and have a characteristic round or cup-shape, while microvesicles are larger, approximately 100 nm–1 *μ*m, and are composed of a round-shaped, heterogeneous population; however, as previously mentioned, a certain degree of size overlap occurs [11, 35, 40, 42, 44–51]. It is important to note that the cup-shape appearance of exosomes visualized by electron microscopy may be an artifact of the fixation process, and their true shape mirrors that of round microvesicles [17]. In addition, there is also some variation in size of EVs based on the method of visualization. For example, exosomes derived from metastatic MDA-MB-231 breast cancer cells were determined to be 40–120 nm in size by SEM and contained the typical exosomal marker CD63 [52]. EVs shed from MDA-MB-231 cells, on the other hand, were larger in size and determined to be 57–440 nm in size with a mean diameter of 121 ± 54 nm by TEM [53]. As another example, tissue factor (TF) antigen has also been identified in breast cancer-derived EVs [53, 54]. 10% of MDA-MB-231-derived MVs were found to express TF, and although EVs up to 220 nm contained active TF, most EVs containing active TF were 100 nm or smaller [53]. In an independent study, TF was instead found in breast cancer MVs 200–350 nm in size. [54]. The term “exosome-like vesicles” (ELV) has been used in some studies to describe vesicles with relatively small size and similar densities, physical characteristics, and protein expression patterns as exosomes [55]. For example, in one study, ELVs from MDA-MB-231 cells were 20–180 nm in size, with the majority being 40–100 nm in size, and contained particles that were both round and cup-shaped [56]. By TEM, ELVs from MCF-7 and MDA-MB-231 cells were reported by Kruger et al. [57] to be 80–200 nm in size and round in shape. For the purpose of this review, ELVs will be referred to as exosomes. Nanoparticle tracking analysis (NTA) is another technique frequently used to determine the concentration and size of EVs [35, 37, 58]. For example, Zheng et al. used NTA to examine Rab27-dependent exosome secretion from MDA-MB-231 cells [37]. NTA analysis determined peak exosome size to be 129 nm, while an average of 79 nm was determined by TEM.

### 2.3. EV Proteins as Cancer Markers

Cancer cell lines are known to secrete significantly more EVs than noncancerous cell lines [59–63]. In line with this, serum from breast carcinoma (BrCa) patients contained significantly higher levels of exosomes than healthy donors [41, 64, 65]. Several EV proteins are differentially expressed in certain stages and types of breast cancer and may be used as diagnostic markers of cancer progression or as a diagnostic marker, that is, “liquid biopsy” for general cancer diagnosis. For example, the oncogenic cancer marker CD24 [66] was uniquely expressed in serum-derived exosomes from BrCa patients [65]. In an independent study, FAK and EGFR, which are overexpressed in cancer [67, 68], were significantly higher in MVs from BrCa patients compared to healthy donors and were found to be differentially expressed depending on the stage of cancer [41]. Survivin-2B, a splice variant of the native protein, which is an apoptosis inhibitor and associated with decreased survivability [69], has also been identified in EVs from BrCa patient serum, and the protein expression is lost with the progression of the disease [70]. In addition to CD24, FAK, EGFR, and Survivin, BrCa serum EVs have been found to contain the disintegrin and metalloprotease ADAM10, as well as the tetraspanin CD9 [41, 65, 70], with the latter being a known functional integrin binding partner in breast cancer cells [71, 72] and a molecular marker of EVs. Similarly to EVs from serum, exosomes from BrCa pleural effusions contained ADAM10, CD9, and CD24 but also contained the epithelial cell adhesion molecule (EpCAM) [65], a highly expressed protein on cancer cells [73]. It should be considered, however, that CD9 is present on EVs of both neoplastic and nonneoplastic origin [74]. HSP70 and Annexin-1, the latter of which regulates apoptosis and inflammation [75], were also identified from BrCa pleural effusion-derived exosomes [65]. In addition to BrCa patient samples, several EV proteins have been identified from immortalized cancer cell lines. In one particular study, the tumor marker extracellular matrix metalloproteinase inducer EMMPRIN [76, 77] was identified on SKBR3 and MCF-7 breast cancer-derived MVs, but not on exosomes [78]. On the other hand, the tetraspanin CD63, a binding partner of integrins and tumor marker whose expression inversely correlates with cancer metastasis [71, 79–84], and tumor susceptibility gene 101 (TSG101), which is a subunit of the endosomal sorting complex required for transport-1 (ESCRT-1) [85], were present almost exclusively on exosomes [78]. Others have reported CD63, ALG-2-interacting protein X (Alix), which is involved in exosome biogenesis and endosomal sorting [86, 87], and TSG101, or a combination of these proteins, from MDA-MB-231, MCF-7, T47D:A18, and Hs-578T breast cancer-derived exosomes [37, 45, 49, 50]. Lysosome-associated membrane glycoprotein 1 (LAMP1) and heat shock cognate 70 (HSC70) were also both found to be in exosomes from MDA-MB-231 cells [56]. Therefore, specific proteins can be used to distinguish breast cancer exosomes from shed MVs.

### 2.4. Proteomic Profiling

There has been extensive proteomic characterization performed on exosomes from breast cancer cells by liquid chromatography-mass spectrometry (LC-MS). This proteomic characterization demonstrated that exosomal content is diverse in nature and varies depending on the cell line from which they originate. They also contain cytosolic as well as membrane proteins involved in signaling pathways and maintenance of cellular structure. In a study performed by Palazzolo et al., LC-MS identified 32 proteins more abundant in the MDA-MB-231 exosomes compared with the cell lysates [56]. These included cytoskeletal proteins such as cytokeratin 9 (K1C9), tropomyosin 4 (TPM4), and transgelin 2 (TAGL2), proteins regulating cell death, including peroxiredoxin 2 (PRDX2), Annexin A5, and heat shock protein 71 (HSP71), and signal proteins such as integrin *α*6 and integrin *α*3 [56]. The latter two proteins are involved in cell migration and therefore play a role in the growth and angiogenesis of tumors [88–93]. Exosomes were also enriched in the beta chain of MHC Class I molecules (B2MG), supporting the involvement of exosomes in altering immune system recognition to promote cancer growth [56]. Consistent with this result, proteomics performed on exosomes from a pleural effusion of a breast cancer patient identified MHC Class I molecules, in addition to B-cell translocation gene 1 (BTG1) and pigment epithelium-derived factor (PEDF) [44]. Since the decrease in tumor surface expression of BTG1 and PEDF has been associated with increased cancer growth and metastasis [94–96], tumor cell exosomal shedding of these proteins obviously decreases the cell surface content of these molecules, which benefits the tumor. Secretome profiling of BT-474 and SKBR3 breast cancer cell lines also showed that exosomes were enriched in proteins associated with antigen processing/presentation, such as heat shock 70 kDa protein 5 (HSPA5), calreticulin (CALR), and proteasome activator complex subunit 2 (PSME2), and proteins associated with glycolysis/gluconeogenesis, such as triosephosphate isomerase 1 (TPI1) and phosphoglycerate mutase 1 (PGAM1) [97]. Proteomics of MCF-7 exosomes showed they contained lipid raft proteins (G protein) and raft-associated proteins (profilin II, HSP27) [36], giving insight into the composition of their exosomal membranes. Others have also documented flotillin-1 and cofilin, proteins associated with lipid rafts in MCF-7 exosomes [45]. Villarreal et al. demonstrated exosomes composed a portion of the MCF-7 and MDA-MB-231 secretomes, and the former included CD63, along with intracellular proteins such as Annexins A2 and A4, and a variety of histones and chaperones [98]. Immunoblotting also showed BT-549 breast cancer exosomes contained Annexins A2 and A6 [99]. A separate study showed BT-549 exosomes were taken up through lipid raft domains, which possibly involves Annexin A2 in the target cell, while Annexin A6 may play a role in the movement of exosomes to the late endosomal compartment [22]. Recently, LC-MS/MS was used to compare proteins of exosomes from both MDA-MB-231 and MCF-7 cells [57]. MDA-MB-231-derived exosomes contained significantly more extracellular matrix proteins than those from MCF-7 cells [57]. MDA-MB-231-derived exosomes also contained more proteins with catalytic activity, while MCF-7 exosomes contained more nucleic acid binding and transport proteins [57]. Annexin A1 was exclusive to MCF-7 exosomes while EpCAM was exclusive to MDA-MB-231-derived exosomes and both contained Annexin A2 and *α*-enolase [57], the latter of which has been shown to promote cancer cell growth, migration, and metastasis [100]. MDA-MB-231- and MCF-7-derived exosomes also differentially expressed certain miRNAs. For example, MDA-MB-231-derived exosomes had higher levels of tumorigenic mir-130a and mir-328, while those from MCF-7 cells had higher levels of mir-301a [57], the expression of which indicates a negative prognosis of patients with invasive ductal or triple negative breast cancer [101, 102]. Therefore, exosomes from both breast cancer cell lines contain proteins and miRNA that are oncogenic in nature, yet they each have their own distinctive identifying markers that may relate to the nature and aggressiveness of the cancer type. Both breast cancer-derived exosomes and MVs contain significant, yet distinctive proteins related to breast cancer progression and metastasis (Table 1). Additional breast cancer-derived exosomal proteins have functional roles in immune evasion and miRNA biogenesis and serve as surrogate metastatic markers (Table 2).

## 3. Visualization of Breast Cancer EVs

Direct visualization of breast cancer EV release and uptake in both live and fixed cells by microscopy has been facilitated by fluorescently tagged EV proteins, fluorescent antibodies or peptides, or lipid fluorescent dyes. In particular, several groups have utilized breast cancer cell lines overexpressing the exosomal marker CD63 with a green fluorescent protein (GFP) fusion tag [22, 46, 103]. A striking example is shown by Suetsugu et al., who engineered MDA-MB-231 and mouse mammary tumor (MMT) cells stably expressing CD63-GFP [103]. From these cell lines, the transfer of CD63-GFP exosomes to nonfluorescent autologous breast cancer cells was visualized* in vitro* and in xenograft models. Upon injection into immunodeficient mice, breast cancer cells expressing CD63-GFP formed tumors that metastasized to the lungs, secreting fluorescent exosomes into both the primary tumor and metastatic microenvironment. Various studies have also utilized PKH dyes, which intercalate with lipids, [32, 52, 104, 105], or fluorescent antibody or peptide markers [43, 106, 107] to stain MVs and exosomes, demonstrating that breast cancer EVs can transfer nucleic acids and proteins to autologous and heterologous cells within the tumor microenvironment, possibly resulting in the acquisition of the cancer phenotypes, favoring tumor progression, immune evasion, and metastasis.

## 4. Horizontal Transmission of miRNA and Proteins

EVs purified from breast cancer cells typically carry specific mRNAs and miRNAs in addition to proteins and can transfer both transcripts and intact proteins to surrounding cancer cells to promote tumor development. In fact, miRNA is enriched in exosomes derived from the breast cancer cell lines 4T.1, MDA-MB-231, and MCF-7 compared to exosomes from normal breast cells MCF10A and NMuMG [64]. Exosomes from metastatic cell lines (MDA-MB-231 and 4T.1) were also enriched in miRNA compared to exosomes from nonmetastatic cells (MCF-7) [64]. miRNAs were found to be secreted into subpopulations of MVs from MDA-MB-231 cells, with different miRNAs packaged into different types of vesicles [48]. Addition of MVs from MDA-MB-231 cells caused an increase in total RNA in human submandibular gland (HSG) cells [108]. In turn, HSG MVs isolated from HSG cells that were treated with MDA-MB-231-derived MVs contained multiple new mRNAs and an increase in protein levels [108]. Cancer cell-derived exosomes are capable of miRNA processing and biogenesis in addition to transfer of miRNA to target cells [64]. This is evidenced by the detection of proteins involved in miRNA biogenesis, including the RISC loading complex (RLC) proteins Dicer, Ago2, and TRBP, in exosomes from breast cancer cell lines and patient samples but not from normal breast cell lines [64].

## 5. Induction of Drug Resistance

Several mechanisms have been described for breast cancer EV-mediated transfer of drug resistance to promote tumor growth and progression. One such mechanism involves the transfer of P-glyoprotein (P-gp), a protein known to be involved in drug resistance [109, 110], through MVs produced from doxorubicin- or docetaxel-resistant breast cancer cells into target endothelial or drug-sensitive cancer cells [111, 112]. These MVs also transferred TrpC5, which caused activation of the NFATc3 transcription factor to stimulate transcription of P-gp mRNA [111]. In addition to the transfer of proteins, the transfer of miRNAs from drug-resistant breast cancer-derived exosomes conferred drug-resistant properties to target cells [47, 104]. Specifically, exosomes from docetaxel-resistant MCF-7 cells contain miRNAs which downregulate mRNA encoding chemosensitive properties when transferred to nonresistant MCF-7 cells [47, 104]. Exosomes from doxorubicin-resistant MCF-7 cells also induced chemoresistance in nonresistant MCF-7 cells through transfer of miRNAs [47]. In another study, exosomes produced from tamoxifen-resistant MCF-7 cells were taken up by MCF-7 wild type cells and released miR-221/222 [32]. miR-221/222 subsequently caused a decrease in P27 and ERa (targets of miR-221/222) protein levels, which caused an increase in tamoxifen resistance in MCF-7 target cells. Besides conferring drug resistance, cancer exosomes can decrease the effectiveness of the therapeutic drug trastuzumab, an antibody that binds HER2 [40]. The presence of HER2 on cancer cells is linked to increased cancer metastasis and tumor proliferation [113, 114]. Exosomes from SKBR3 and BT-474, both of which are HER2-overexpressing breast cancer cell lines, contain active HER2 and, along with exosomes from HER2-positive breast cancer patient serum, bound to trastuzumab to decrease the drugs' effectiveness at inhibiting SKBR3 cell proliferation [40]. Treatment of BT474 cells with epidermal growth factor (EGF) and heregulin (HRG), which are growth factors secreted by cancer cells [115–117] and activators of HER2 [118, 119], caused a significant increase in exosome production [40], leading to the possibility that enhanced secretion of exosomes may be a way for the tumor microenvironment to support cancer growth in the presence of therapeutic agents. Consistent with these results, HER2^+^ exosomes from BT-474 and SKBR3 cells decreased the trastuzumab-induced cytotoxic effects of peripheral blood mononuclear cells (PBMC) against BT-474 cells [120]. Besides the transfer of proteins and miRNA to cancer cells, breast cancer EVs can also directly secrete the anticancer drug doxorubicin [121]. The number of secreted MCF-7-derived EVs positively correlated with increasing drug concentration, which suggested EV release is a direct mechanism for imparting chemoresistance within a tumor [121].

## 6. Therapeutic Implications of EVs

It is possible to take advantage of the drug delivery capabilities of exosomes for breast cancer therapy, as shown by Tian et al. [122]. Doxorubicin, incorporated into exosomes from immature dendritic cells (imDC) and taken up by both MDA-MB-231 and MCF-7 cells, caused an inhibition of cell proliferation. When injected into nude mice implanted with MDA-MB-231 tumors, it caused a reduction in tumor growth [122]. Specific targeting to tumors was achieved by incorporating an RGD integrin target sequence in the exosomes which bound to MDA-MB-231 cells, which naturally express high levels of integrin *α*v [123]. Exosomes from HEK293 cells stably expressing EGF or EGFR peptide have also been studied as therapeutic agents by targeting breast cancer cells expressing high levels of EGFR (such as the HCC70 breast carcinoma cell line) [39]. The tumor suppressor let-7a miRNA was transfected into HEK293 cells expressing EGFR peptide, and exosomes produced from these cells significantly decreased HCC70 tumor growth in mice [39]. Additionally, treatment of 4T.1 murine breast cancer cells with epigallocatechin gallate (EGCG), a chemical with antitumor effects, caused an increase in the levels of the tumor suppressor miR-16 within exosomes [124]. Exosomes from EGCG-treated breast cancer cells subsequently decreased NF*κβ* activity and M2 polarization in tumor-associated macrophages (TAM) through miR-16 [124]. M2 macrophages, which inhibit inflammation during the end stages of inflammatory processes such as wound healing [125], are known to have tumor-promoting phenotypes [126, 127]. Consequently, therapeutic agents can induce transfer of tumor suppressors to surrounding macrophages through exosomes to decrease their cancer-associated properties.

## 7. EVs as Immunomodulators

**Figure 1:**
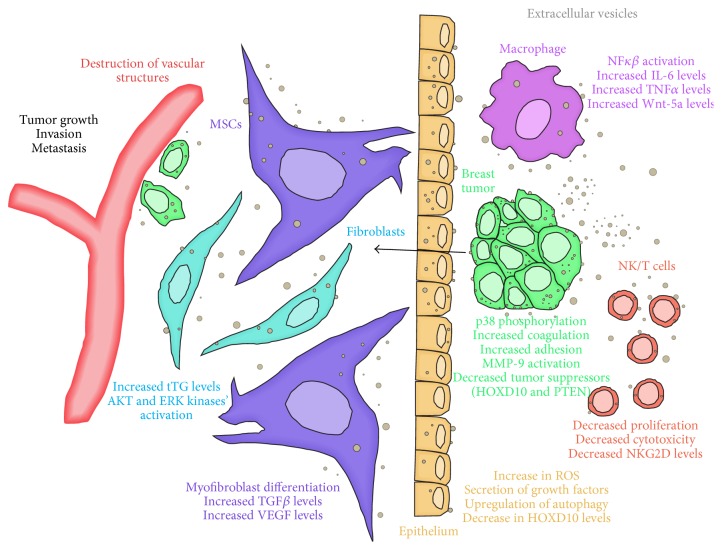
Schematic of the numerous interactions between breast tumor-derived EVs and stromal, epithelial, and immune cells within the tumor niche and the resulting changes that enhance cancer growth and metastasis. Breast cancer cells avoid detection from the immune system through EV-mediated decrease in the cytotoxicity of NK and T-cells and secretion of proinflammatory cytokines from macrophages. Tumor EVs enter fibroblasts and stimulate proangiogenic ERK and AKT kinase activation. Concurrently, EVs cause the secretion of oncogenic cytokines TGF*β* and VEGF in mesenchymal stem cells. Additionally, tumor EVs enter surrounding cancer cells to upregulate signaling pathways that promote growth and metastasis, including increases in coagulation, adhesion, and a decrease in levels of tumor suppressors. To promote invasion, EVs induce epithelial cells to secrete growth factors and cause destruction of endothelial vascular structures, thus enhancing tumor growth.

Many groups have studied the immunosuppressive effects of tumor-derived EVs and their relation to cancer progression within the tumor microenvironment (Figure 1). In one study, MVs from 36% of breast cancer patients contained indoleamine 2,3-dioxygenase (IDO), an enzyme that has a role in immunosuppression and tumor survival [128]. Another example of exosomal-mediated immune modulation was described in a study involving TS/A murine mammary tumor-derived exosomes, which were found to act as immune system suppressors to promote tumor progression by inhibiting the differentiation of myeloid precursors in the bone marrow into dendritic cells, partially through induction of IL-6 mRNA [129]. Myeloid-derived suppressor cells (MDSCs) are involved in tumor growth, partially through the inhibition of T-cell activation via Toll-like receptor (TLR) adaptor protein MyD88 [130]. Murine melanoma-derived exosomes were found to exacerbate the effects of MDSCs, which included the induction of the proinflammatory cytokine CCL2 through MyD88 [131]. This same study also discovered that 4T.1-derived exosomes caused an increase in lung metastases through CCL2 [131]. Similarly, breast cancer TS/A-derived exosomes injected into a mouse model caused an increase of MDSCs within both the primary tumor and the spleen [132]. Breast tumor-derived exosomes have also been found to reduce the immune response by inhibiting NK cytotoxicity. In one case, TS/A and 4T.1 breast tumor-derived exosomes inhibited activated NK cell proliferation and prevented them from killing TS/A and 4T.1 tumor cells [133].

In another study, injection of TS/A tumor-derived exosomes into the general circulation of mice decreased the cytotoxicity of NK cells by reducing the percentage and activity of NK cells [105]. TS/A-, 4T.1-, and MDA-MB-231-derived exosomes also decreased stimulated NK cell proliferation, and mice implanted with TS/A or 4T.1 tumors and treated with TS/A or 4T.1 breast tumor-derived exosomes had more rapid tumor growth and metastases compared to controls [105]. The human MHC Class I chain-related genes MICA and MICB are ligands of the NKG2D receptor and stimulate the immune response by activating T-cells [134–137]. To evade detection by the immune system, MDA-MB-231-derived exosomes lack MICA or MICB and therefore there is no immune stimulation [138]. Furthermore, ADAM17 in MDA-MB-231 breast cancer cells cleaves MICA/MICB from the cell surface of the cancer cell to produce a soluble form, sMICA/sMICB, which may result in a type of immune decoy [138]. Exosomes from T47D breast carcinoma cells decreased the amount of NKG2D-positive lymphocytes within peripheral blood lymphocytes (PBL), which was modulated through NKG2D ligands such as MICB, CD81, and MHC Class I on exosomes [33]. These T47D-derived exosomes also led to inhibition of the cytotoxic function of CD8^+^ T-cells, which may be related to the downregulation of NKG2D [33]. This could be yet another mechanism for cancer to evade and/or inhibit the host immune system. A study by Deng et al. showed exosomes isolated from the 4T.1 breast cancer cell line that had interacted with immunosuppressive leukocytes from the tumor microenvironment conferred properties that enhanced tumor metastasis through production of MMP-9 and proinflammatory cytokines [139]. One interesting study suggested how the structural interactions of MDA-MB-231- and MCF-7-derived MVs with human beta-defensin 6 (hBD6), a class of proteins with antimicrobial activity [140], were linked to metastatic potential [141]. There were more nuclear magnetic resonance (NMR) chemical shifts and larger resonance intensity ratios associated with MCF-7-derived MVs compared to MDA-MB-231-derived MVs upon association with hBD6, which indicated stronger binding to the MCF-7-derived MVs [141]. This group proposed the weaker interaction of MDA-MB-231-derived MVs with hBD6 could suggest a greater capability of evading immune detection and one explanation for the increased metastatic properties of this cell line [141].

## 8. EV Contribution to Breast Tumor Growth and Metastasis

### 8.1. EV Transfer of Cancerous Phenotypes

**Figure 2:**
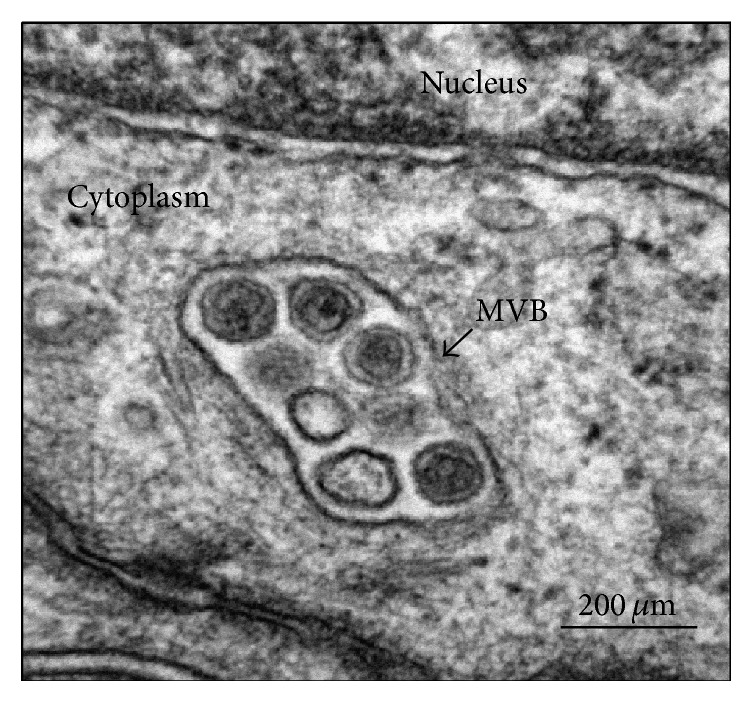
Early biogenesis of exosomes present within a multivesicular body (MBV) of the MARY-X spheroids is depicted. The MBV will subsequently fuse with the plasma membrane and exosomes will be released from the cell.

As mentioned above, cancer cell-derived EVs contain a number of oncogenic proteins and miRNAs, which can be transported to surrounding cancer cells to elicit phenotypic changes within target cells. Several groups have investigated the mechanisms through which breast tumor-derived EVs upregulate prometastatic pathways after entering nearby cancer cells. One scenario involves breast cancer MVs increasing cancer cell invasion through extracellular matrix metalloproteinase inducer- (EMPRINN-) dependent phosphorylation of p38 [42]. MDA-MB-231-derived MVs also contain RhoA and RhoC GTPases, which are upregulated in cancer and involved in invasion and metastasis [142, 143], as well as ADP-ribosylation factor 1 (ARF1), which is associated with MV release and MMP-9 activity within MVs [144]. MMP-9 is involved in the degradation of extracellular matrix, a critical step in cancer cell invasion [145, 146]. MDA-MB-231-derived MVs have also been reported to transfer TF to MCF-7 cells to cause increase in procoagulant activity [147], which has been correlated with increased metastatic capabilities [148]. Cellular adhesion is also known to be important in the growth and metastatic progression of cancer cells [149, 150]. To this end, the uptake of exosomes derived from BT-549 cells into target cancer cells caused increased cellular adhesion and spreading through histone H2A binding to heparin sulfate proteoglycans [46]. Another study by Melo et al. [64] highlighted the importance of exosomal miRNA processing in tumor growth. This group showed that breast cancer exosomes have all the components required for cell-independent processing of pre-miRNAs into mature miRNAs. By treating normal breast cells with exosomes from metastatic breast cancer cells and measuring miRNA changes within the target cell, they showed that exosomal miRNAs have the ability to alter the transcriptome of target cells. Similarly, treatment of MCF10A cells with MDA-MB-231-derived exosomes led to upregulation of miRNAs involved in cancer metastasis, resulting in downregulation of proteins including PTEN and HOXD10, as well as increases in MCF-710A viability, proliferation, and colony formation, all processes in which the capability of miRNA biogenesis was essential. MDA-MB-231-derived exosomes injected with MCF10A cells also caused tumor growth in nude mice, which was decreased when miRNA biogenesis within exosomes was inhibited [64]. Therefore, cancer growth is influenced by miRNA biogenesis in exosomes. In addition to miRNA, exosomes can also transfer cancerous properties to heterologous cancer cells to promote tumor aggressiveness and metastasis. O'Brien et al. demonstrated that exosomes isolated from an invasive variant of triple negative breast cancer (TNBC) cells (Hs578Ts_(i)8_) caused an increase in the growth rate, migration, and invasive capacity of SKBR3, MDA-MB-231, and HCC1954 breast cancer cell lines, as well as causing an increase in endothelial cell angiogenesis [50]. Galindo-Hernandez et al. reported that an epithelial-mesenchymal transition-like process was induced in MCF10A cells by EVs isolated from plasma of women with breast carcinoma [151]. Exosomes from the serum of patients with TNBC also produced significantly greater invasion of breast cancer cell lines compared to exosomes from healthy donors [50]. Recently, using a TNBC xenograft model of inflammatory breast cancer (MARY-X) [152], a model which forms tight tumor cell aggregates (termed spheroids)* in vitro*, which represent the* in vivo* equivalent of lymphovascular tumor emboli, complete exosomal biogenesis could be observed by TEM (Figure 2). In this model, large numbers of secreted exosomes observed within the intercellular space between cancer cells suggest the possibility that important exosomal-mediated autocrine signaling occurs in actual tumor emboli which may give them a survival advantage (unpublished observations). Since most breast cancers are adenocarcinomas, which are present in both metastatic sites and the circulation as circulating tumor cells (CTCs), this exosomal-mediated autocrine signaling may have wide therapeutic implications.

### 8.2. Hypoxic Effects on EV-Influenced Metastasis

Cancer cells have a unique ability to survive and grow under hypoxic conditions [153]. Exosome secretion plays a role in the ability of tumors to metastasize under limited oxygen conditions. Hypoxic conditions are known to increase MV and exosome release from a variety of breast cancer cell lines, including MCF-7, MDA-MB-231, and SK-BR3 [43, 45], which requires hypoxia-inducible factor- (HIF-) dependent RAB22A expression [43]. Hypoxic breast cancer-derived MVs caused increases in tumor metastasis and invasion, which were dependent on the expression of RAB22A within the MVs [43]. A low pH often accompanies hypoxia in cancerous phenotypes [154], and it has been previously reported that melanoma-derived exosome secretion and uptake increases with decreased pH levels [24]. Additional studies showed that the MCF-7 breast cancer cell line became more resistant to the chemotherapeutic drug cisplatin at lower pH levels [155], and treatment of metastatic breast cancer patients with proton-pump inhibitors (PPI) led to enhanced efficacy of chemotherapy [156]. From these results, it can be proposed that treatment of breast carcinomas with PPIs could lead to decreased exosome number within the tumor microenvironment, leading to enhanced chemosensitivity. Breast cancer-derived exosomes produced under hypoxic conditions can also promote inflammatory cross talk within the tumor niche to promote growth, which is decreased by activation of nuclear receptors within the cancer cell [157]. This leads to the possibility of nuclear receptors as new therapeutic targets, whose efficacy can be increased by taking advantage of the exosome-mediated delivery system between cancer cells.

### 8.3. Effects on the Tumor/Metastatic Niche

Breast tumor exosomes have the ability to prime the premetastatic niche, which involves the transfer of proteins and activation of signaling pathways within epithelial cells, stromal cells, macrophages, and fibroblasts that promote tumor progression (Figure 1). Several studies have demonstrated that exosomes from cancer cells have this effect on the surrounding microenvironment. MVs derived from MDA-MB-231 have been shown to confer cancerous phenotypes to NIH 3T3 fibroblasts as well as noncancerous MCF10A human breast epithelial cells, including increased survival and cellular transformation, partly through the transfer of tissue transglutaminase (tTG) [107]. MDA-MB-231-derived MVs caused NIH 3T3 tumor growth in nude mice through activation of AKT and ERK kinases in NIH 3T3 fibroblast cells, requiring tTG [107]. In another example, MDA-MB-231-, T47DA18-, and MCF-7-derived exosomes were taken up by human primary mammary epithelial cells (HMEC), causing an increase in reactive oxygen species (ROS) and secretion of tumor growth factors from HMECs [49]. Increases in ROS by breast cancer-derived exosomes led to upregulation of autophagy through an increase in the phosphorylation of DNA damage response proteins (DDR) (ATM, H2AX, and Chk1) and p53 stabilization [49]. Autophagy, which is the internalization of damaged material within a cell, is an important survival mechanism for cancer cells to acquire energy to keep up with the demands of tumor growth [158]. In addition, cancer exosomes caused an increase in HMEC autophagy in part by transferring LC3 proteins, involved in autophagosome cargo sorting [159], to HMEC cells [49]. A study done by Cho et al. indicates that breast tumor-derived exosomes can differentiate mesenchymal stem cells (MSC) into tumor-associated myofibroblasts [160], which are known to contribute to tumor growth, progression, and metastasis within the tumor microenvironment [161]. In particular, MDA-MB-231- and MCF-7-derived exosomes increased the expression of *α*-SMA, which is an indicator of myofibroblasts, through the activation of SMAD2-dependent pathways, and cytokines such as TGF*β* and VEGF, which support tumor growth, when added to adipose-derived mesenchymal stem cells (ADSC) [160]. Metastatic enhancement of breast cancer cells is regulated through exosomal miR-105, which can target tight junctions in primary human microvascular endothelial cells (HMVEC), resulting in the increased permeability of cell monolayers, the destruction of vascular structures, and induced HMVEC migration [162]. Additionally, treatment of HMVECs with MDA-MB-231-derived exosomes increased transendothelial migration of MDA-MB-231 cells through HMVEC monolayer, and mice pretreated with MDA-MB-231-derived exosomes and then injected with MDA-MB-231 cells had increased lung and brain metastases [162]. MDA-MB-231-derived exosomes can also transfer miR-10b, which is more highly expressed in exosomes from metastatic as opposed to nonmetastatic cell lines, to immortalized human mammary epithelial (HMLE) cells to reduce levels of the tumor suppressor HOXD10 [163] protein and subsequently increase cell invasion [52]. Inflammation from macrophages promotes tumor growth and metastasis to distant sites [164, 165]. Concurrently, macrophages treated with breast cancer-derived exosomes caused significant increases in NF-*κβ* activation and production of inflammatory cytokines (including IL-6 and TNF*α*) compared to exosomes from normal breast cells through exosomal binding to the Toll-like receptor 2 (TLR2) [106]. Additionally, MDA-MB-231 cells transplanted into immunodeficient mice released exosomes that entered distant macrophages in lymph nodes, causing increases in IL-6 expression [106]. This showed breast cancer exosomes can alter macrophage phenotypes to cause an increase in metastasis. In another study, MCF-7-derived EVs transferred Wnt 5a mRNA, which plays an important role in tumor invasion, to macrophages, where it was translated into Wnt 5a protein [78]. This Wnt 5a protein in macrophages was then packaged into EVs and secreted from cells. The macrophage EVs containing Wnt 5a subsequently caused an increase in MCF-7 cell invasion [78]. Therefore, breast cancer EVs can transfer mRNA to macrophages, which can, in turn, translate and package cancerous proteins into vesicles, which are then subsequently secreted into the tumor microenvironment to enhance the invasive capacity of surrounding cancer cells in a paracrine manner.

## 9. Conclusions

There have been many proteins identified within breast cancer EVs that give insight into the nature and severity of the disease. These could serve as possible diagnostic markers to be used in conjunction with current analytical techniques. Both exosomes and MVs are key players in the progression of cancer and elicit a multitude of cellular responses within their target cells, at both the mRNA and protein levels. There are multiple mechanisms by which cancer cells avoid immune system recognition through EVs, such as secretion of immunosuppressive proteins, inhibition of NK cell proliferation, or a decrease in CD8^+^ T-cell cytotoxicity. Some targeted breast cancer therapies have been investigated employing exosomes as a vehicle for drug delivery, but due to the complex nature of EV-cell interactions, additional studies need to be performed to achieve a therapeutic strategy with a favorable therapeutic index using an exosome approach. Tumors have the unique ability to support their progression by using MVs and exosomes to deliver procancerous transcripts and proteins, to both other cancer cells and nontransformed cells. EVs are key players within the cancer niche and are able to thrive in the acidic, hypoxic environments common to tumors in order to confer prometastatic phenotypes such as inflammation, migration, and invasion. While there have been many advances in recent years, future investigations involving EVs and their contribution to tumor growth are crucial to the continued understanding of cancer development.

## Figures and Tables

**Table 1:** Comparison of breast cancer-derived MV and exosomal proteins.

Role	Microvesicles	Exosomes
Extracellular matrix degradation	EMPRINN [42, 78] ARF1 [144]	ADAM10 [51, 65]

Cancer invasion/metastasis	RhoA/RhoC GTPase [144] FAK [41] EGFR [41] TF [53, 54, 147]	Annexins A2, A5, and A6 [55, 56, 97, 98] EpCAM [57, 65, 120] H2A [46] CD24 [65] HSP70 [36, 65] *α*-enolase [57] Integrins *α*3, *α*6 [56]

Cell survival	IDO [128] tTG [107]	PRDX2 [56] HSC70 [56]

**Table 2:** Additional breast cancer-derived exosomal proteins and their functional roles.

Role	Exosomes
Immune evasion	CALR [97] MHC Class I molecules [33, 37, 44, 56] PSME2 [97]

miRNA biogenesis	Dicer [64] Ago2 [64] TRBP [64]

Integrin binding/signaling partner	CD63 [45, 49, 50, 52, 64, 78, 98, 139] CD9 [45, 51, 64, 65, 133, 138, 139]
